# Diaphragm ultrasound and diaphragmatic 2D speckle tracking imaging in acute heart failure: a case series

**DOI:** 10.1093/ehjcr/ytae632

**Published:** 2024-11-28

**Authors:** Abdallah Fayssoil, Pierre Boisson De Chazournes, Marie Hauguel-Moreau, Arnaud Mansart, Nicolas Mansencal

**Affiliations:** Department of Cardiology, Ambroise Paré Hospital, Assistance Publique-Hôpitaux de Paris (AP-HP), Centre de référence des cardiomyopathies et des troubles du rythme cardiaque héréditaires ou rares, Université de Versailles-Saint Quentin (UVSQ), Boulogne-Billancourt, France; Department of Cardiology, Ambroise Paré Hospital, Assistance Publique-Hôpitaux de Paris (AP-HP), Centre de référence des cardiomyopathies et des troubles du rythme cardiaque héréditaires ou rares, Université de Versailles-Saint Quentin (UVSQ), Boulogne-Billancourt, France; Department of Cardiology, Ambroise Paré Hospital, Assistance Publique-Hôpitaux de Paris (AP-HP), Centre de référence des cardiomyopathies et des troubles du rythme cardiaque héréditaires ou rares, Université de Versailles-Saint Quentin (UVSQ), Boulogne-Billancourt, France; Université Paris-Saclay, UVSQ, Inserm, 2I, 78000 Versailles, Yvelines, France; Department of Cardiology, Ambroise Paré Hospital, Assistance Publique-Hôpitaux de Paris (AP-HP), Centre de référence des cardiomyopathies et des troubles du rythme cardiaque héréditaires ou rares, Université de Versailles-Saint Quentin (UVSQ), Boulogne-Billancourt, France

**Keywords:** Diaphragm, Ultrasound, Heart failure, 2D strain imaging, Case report

## Abstract

**Background:**

Respiratory muscle function can be affected in patients with heart failure. Ultrasound can be used to assess diaphragm, the main inspiratory muscle. Speckle tracking imaging is an imaging technology providing the evaluation of tissue deformation during contraction. We aimed to evaluate the contribution of traditional echography and 2D speckle tracking imaging in the evaluation and monitoring of patients with acute heart failure (AHF).

**Case summary:**

We report a series of four cases of AHF. Diaphragm ultrasound coupled with diaphragm 2D speckle tracking imaging was performed at admission and after decongestive therapy, in cardiac intensive care unit. Patients, at admission, disclosed higher diaphragm 2D strain value and higher diaphragm inspiratory motion value in the context of higher cardiac loading that significantly decrease after decongestive therapy, except for one patient. Diaphragm motion remained less than 10 mm (weakness), despite medical therapy in Cases 2, 3, and 4. Among them, 3 months later, one patient (Case 3) experienced an episode of AHF.

**Discussion:**

Diaphragm ultrasound coupled with diaphragm 2D speckle tracking imaging is feasible and may be used to monitor respiratory status patients with AHF.

**Learning points**

- Diaphragm function can be evaluated using ultrasound, by the measurement of diaphragm motion, from subcostal view.
- Diaphragm motion and diaphragm 2D strain value may be affected by cardiac loading, and ultrasound can be used to monitor diaphragm function during clinical management.

## Introduction

Chronic heart failure (CHF) disrupts respiratory muscle function, thereby contributing to reduced exercise capacity.^1^ Approximately 40%–50% of patients with heart failure (HF) experience inspiratory muscle weakness, which is associated with poor outcomes.^2,3^ The diaphragm, which is the main inspiratory muscle, can be analysed using ultrasound.^4^ Recently, two-dimensional (2D) speckle tracking imaging emerged as a potential technique for assessing diaphragm.^5,6,7^ This study aimed to evaluate the feasibility of 2D speckle tracking imaging for diaphragm evaluation and the contribution of ultrasound and 2D speckle tracking imaging to diaphragm function assessment in patients with acute heart failure (AHF).

**Figure 1:**
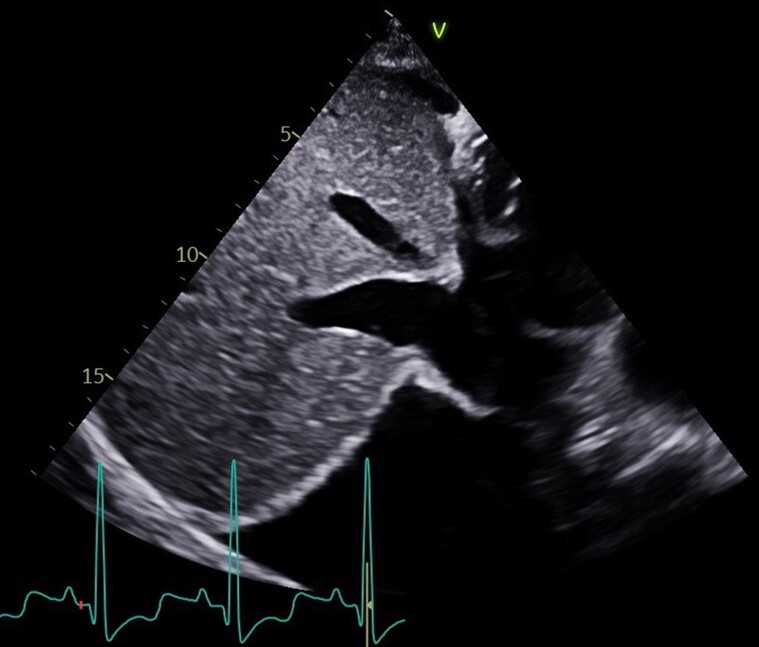
Two-dimensional diaphragm ultrasound showing the diaphragm region from the subcostal view used for two-dimensional strain imaging analysis. The bright line surrounding the liver indicates the diaphragm.

**Figure 2:**
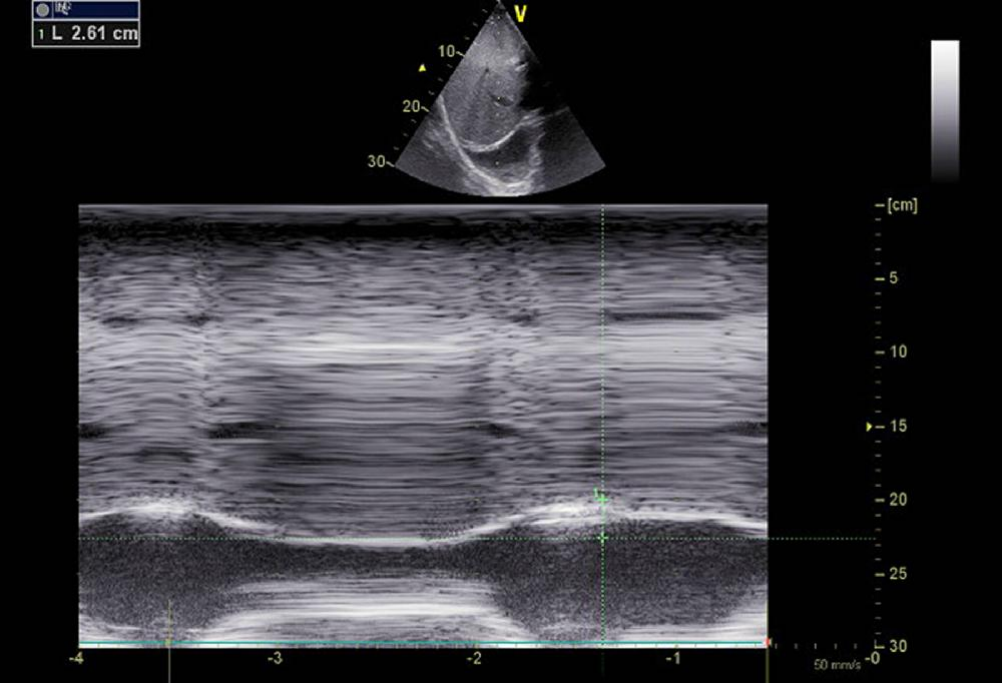
Measurement of the diaphragm motion from the subcostal view. The M-mode was applied perpendicularly to the diaphragm. During inspiration, the diaphragm exhibited physiological caudal displacement. The diaphragm excursion was at 26 mm.

We focused the ultrasound analysis on the right hemidiaphragm for practical purposes. Diaphragmatic inspiratory motion was recorded using VIVID S70 (GE Medical Systems, Horten, Norway), with the cardiac probe M5Sc-D (1.5–4.6 MHz) positioned between the midclavicular line and the anteroaxillary line, at the subcostal view. The patient was placed on semirecumbent position. The operator visualized the right diaphragm as a bright line covering the liver (*Figure 1*; Supplementary material online, Video S1). M-mode was then applied perpendicularly to the hemidiaphragm, providing the diaphragm excursion (DE) measurement.^4^ The normal diaphragm motion measures more than 10 mm.^8^*Figure 2* illustrates the diaphragm motion measurement from the subcostal view *via* ultrasound.

**Figure 3:**
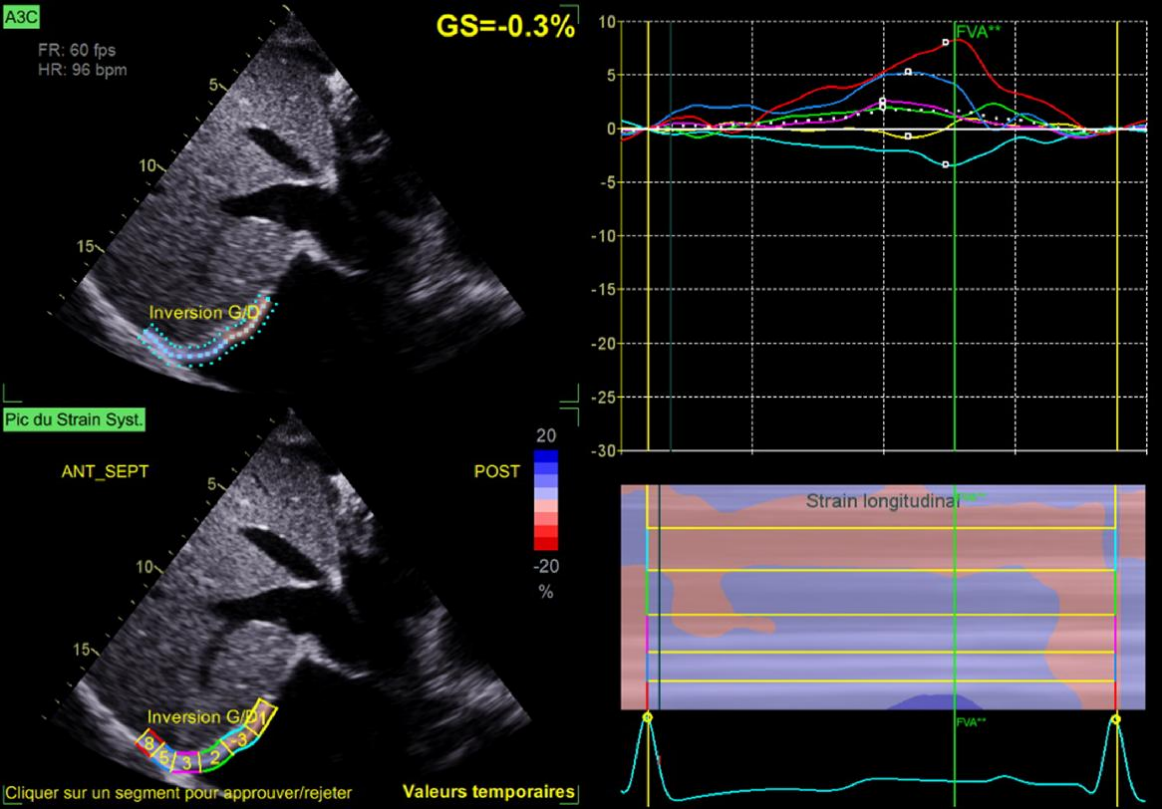
Two-dimensional diaphragmatic strain imaging of advanced acute heart failure with vena cava dilation.

For strain analysis, diaphragm cine-loops were recorded from the subcostal view, using three consecutive cycles, and were adjusted to include at least a total breathing cycle, using a frame rate of at least 40 frames/s. The reference point for image analysis was the R wave onset. We adjusted the region of interest manually to include the diaphragm muscle (*Figure 1*). Speckle tracking imaging has been validated to assess diaphragm function.^5,6,7^ From the subcostal view, at quiet breathing, the normal diaphragm strain value was −2% and reached −4.6% at forced breathing.^7^*Figure 3* and Supplementary material online, Video S2, show the 2D diaphragmatic strain imaging of advanced HF. The diaphragmatic strain value increases as the inspiratory loading increases; this value correlates with transdiaphragmatic pressure.^5^

In our Echo lab, the intraoperator reproducibility of 2D diaphragmatic strain imaging was evaluated in 26 patients. To test the intraoperator agreement (reproducibility) of ultrasound measurements, the observer performed measurements twice at 2 min apart. The mean variability coefficient was 14.5 (standard deviation, 10.4), and the Kendall concordance coefficient was higher (0.98), with a *P*-value of 0.003.

## Summary figure

**Figure:**
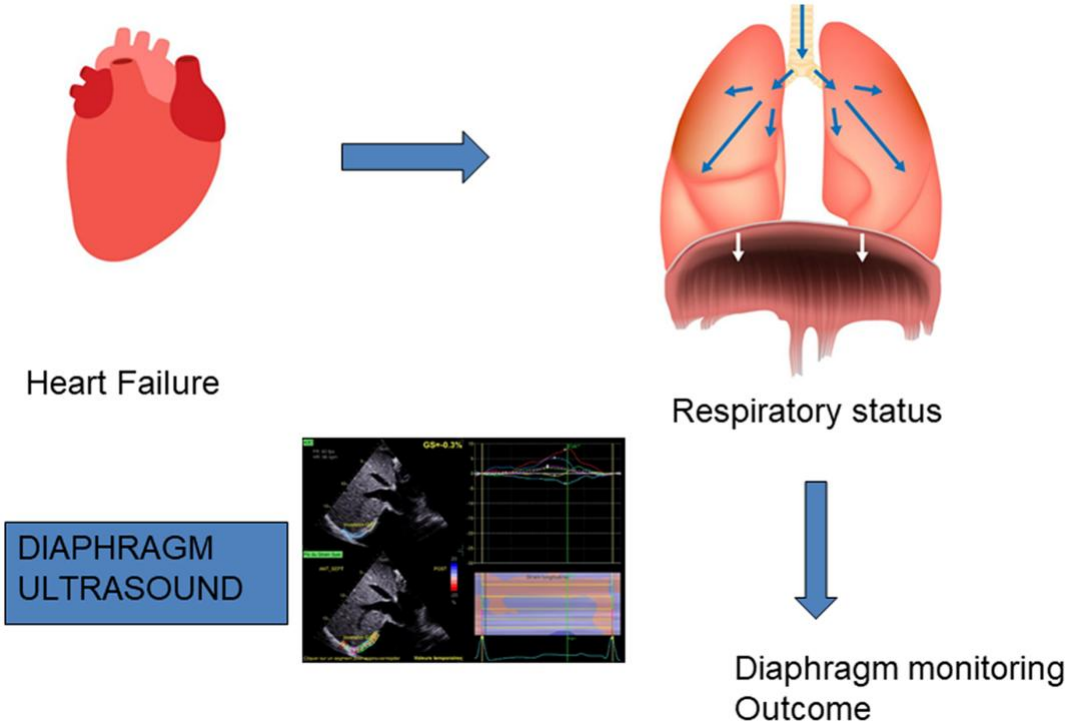


## Patient 1

**Figure 4:**
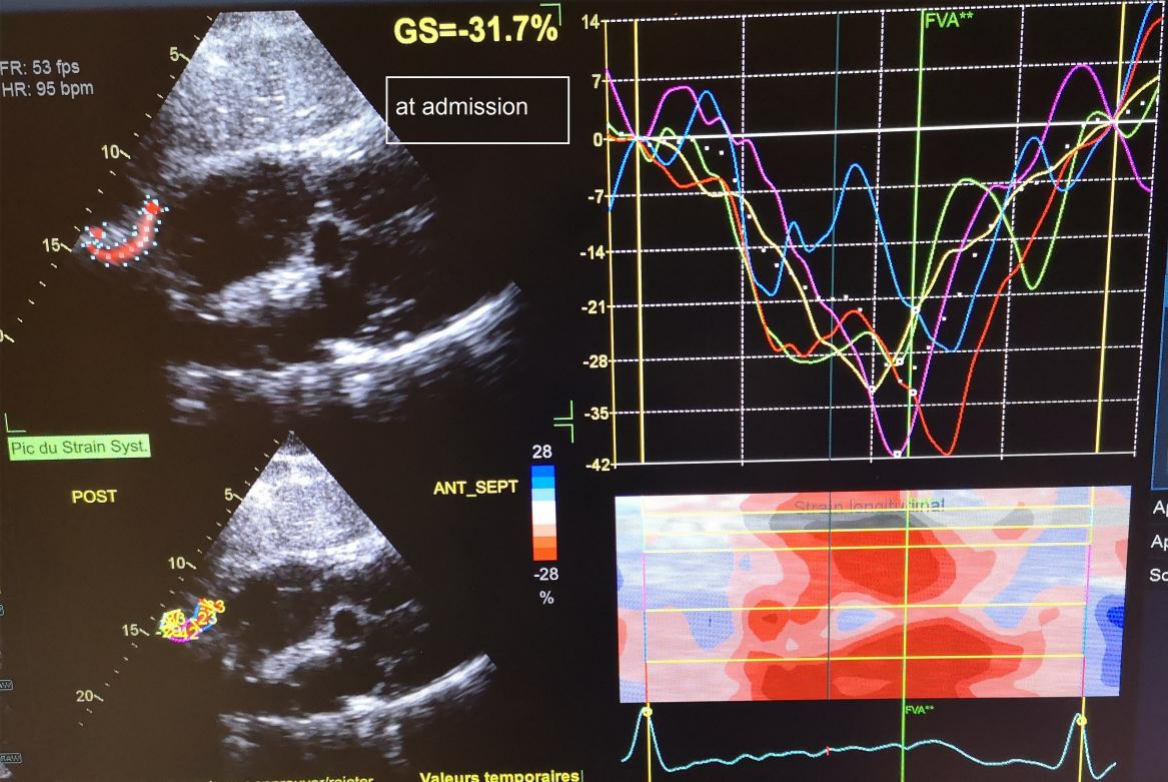
Two-dimensional diaphragmatic strain imaging in a patient with acute heart failure at admission (*Patient 1*).

**Figure 5:**
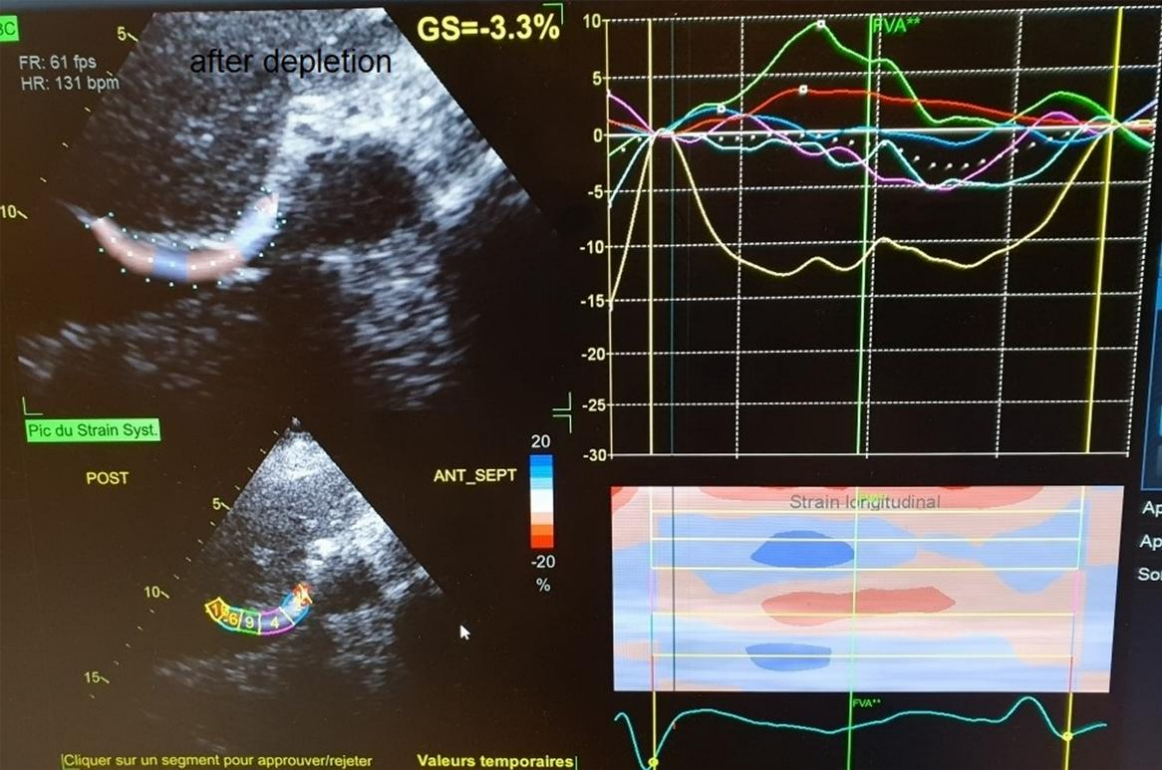
Two-dimensional diaphragmatic strain imaging in a patient with acute heart failure after decongestive therapy and non-invasive ventilation (*Patient 1*).

An 88-year-old woman was admitted to the cardiac critical intensive care unit (CICU) because of ARF. Her past medical history included diffuse interstitial pneumonia and undernutrition. She took daily loop diuretics and antiplatelet drugs. At admission, her body mass index (BMI) was 16.9 kg/m^2^. Clinical examination revealed blood pressure (BP), 128/40 mmHg; heart rate (HR), 91 b.p.m.; leg oedema; and lung crackles. Transcutaneous oxygen saturation (SaO2) was 88% with oxygen therapy (9 L/min), and the respiratory rate (RR) was 41 breaths/min. Her laboratory results were as follows: N-terminal pro-brain natriuretic peptide (NT-proBNP), 15 436 ng/L (normal ranges < 125 ng/L); haemoglobin, 12.9 g/dL (normal ranges 13–17 g/dL); and creatininemia, 80 µmol/L (normal ranges 59–104 µmol/L). The blood gas exchange (BGE) results were the following: pH, 7.37 (normal range 7.38–7.42); PCO_2_, 35 mmHg (normal range 35–45 mmHg); PO_2_, 117 mmHg (normal ranges > 90 mmHg); and bicarbonates, 20.4 mmol/L (normal ranges 22–26 mmol/L). Echocardiography disclosed right ventricular (RV) systolic dysfunction (peak St RV, 9 cm/s), RV dilation with interventricular septum flattening, association with high systolic pulmonary arterial hypertension (sPAP, 60 mmHg), and inferior vena cava non-compliance (diameter, 18 mm/18 mm). Thoracic computed tomography (CT) scan showed a ground glass opacity without pulmonary embolism. The 2D diaphragmatic strain imaging revealed a diaphragm strain value of −32% (Supplementary material online, Video S3), which reduced to −3% (*Figures 4* and *5*) 12 h later, after loop diuretic and non-invasive ventilation (NIV). At Day 4, the respiratory status of the patient worsened requiring admission in ICU. The patient died after 5 weeks of follow-up.

## Patient 2

An 85-year-old male patient was admitted to the CICU because of AHF. His past medical history included systemic arterial hypertension, paroxysmal atrial fibrillation (AF), pacemaker implantation, and peripheral artery disease. This patient took loop diuretics, beta-blockers, angiotensin-converting enzyme inhibitors, calcium channel blockers, and anticoagulants daily. At admission, his BMI was 28 kg/m^2^, and the BP was 120/80 mmHg. Clinical examination revealed acute rest dyspnoea with thoraco-abdominal swing and lung crackles; high oxygen therapy was introduced (15 L/min). The BGE revealed the following: pH, 7.33; PCO_2_, 47 mmHg; PO_2_, 56 mmHg; and bicarbonates, 25 mmol/L. Furthermore, the laboratory results were as follows: NT-proBNP, 3401 ng/L; creatininemia (174 µmol/L); haemoglobin, 10.8 g/dL; and normal C-reactive protein (CRP) levels (normal range < 5 mg/L). Chest CT scan revealed bilateral pleural effusion and lung oedema. Echocardiography disclosed concentric left ventricular (LV) hypertrophy, preserved LV ejection fraction (LVEF), and an increased filling pressure with LA size dilation (41 mL/m^2^), tissue Doppler peak lateral e′ velocity at 9 cm/s, maximal peak velocity tricuspid regurgitation at 3.2 m/s, sPAP at 55 mmHg, and inferior vena cava dilation (28 mm). Diaphragm ultrasound was performed while the patient had no NIV. The diaphragm 2D strain value was +50%, whereas the DE was 51 mm. After loop diuretic therapy and NIV, the second ultrasound, which was performed 12 h later without NIV, revealed a diaphragm 2D strain value of −1% with a DE of 7 mm. At Day 12, the patient was discharged at a rehabilitation care. After a 6-month follow-up, the patient did not experience any hospitalization for HF.

## Patient 3

An 86-year-old male patient with AHF was admitted to the CICU. His past medical history included AF, systemic arterial hypertension, dyslipidaemia, mitral bioprosthesis, tricuspid valve annuloplasty, and CHF. He suffered from acute rest dyspnoea. The patient took calcium channel blockers, angiotensin II receptor antagonists (ARA-II), loop diuretics, gliflozin, statin, and oral anticoagulants. The BMI was 25 kg/m^2^. At admission, clinical examination revealed BP, 150/67 mmHg; HR, 98 b.p.m.; acute rest dyspnoea with lung crackles; SaO2, 81% (using accessory muscles); and RR, 40/min. Therefore, oxygen therapy (9 L/min) was introduced, providing a SaO2 of 95%. Laboratory results were as follows: haemoglobin, 12.7 g/dL; creatininemia, 87 µmol/L; and CRP, 17 mg/L. The BGE revealed the following: pH, 7.25; PCO_2_, 42 mmHg; PO_2_, 58 mmHg; and bicarbonates, 22 mmol/L. Additionally, NT-proBNP was 2884 ng/L. In chest radiography, lung oedema was detected. The echocardiography disclosed LV diastolic dysfunction with an increase of filling LV pressure with LA size dilation (54 mL/m^2^), tissue Doppler peak lateral e′ velocity at 8 cm/s, peak septal e′ velocity at 2 cm/s, maximal peak velocity tricuspid regurgitation velocity at >2.8 m/s, an LVEF of >50%, an St RV value at 7 cm/s, a tricuspid annular plane systolic excursion (TAPSE) of 8 mm, sPAP at 58 mmHg, and inferior vena cava dilation (21 mm). Moreover, diaphragm ultrasound disclosed a reduced diaphragm inspiratory motion (6 mm). The patient was treated using loop diuretics and NIV. The introduction of NIV provided diaphragm motion increase immediately (14 mm). One hour after the NIV removal, the second diaphragm ultrasound showed a diaphragm motion at 5 mm and a 2D strain diaphragmatic ultrasound of −2.4%. On Day 7, the patient was discharged home. The patient was re-admitted in cardiology 3 months later because of AHF.

## Patient 4

A 52-year-old patient with AHF was admitted to the CICU. The patient suffered from subacute rest dyspnoea. Clinical examination revealed BMI 29 kg/m^2^, BP 121/91 mmHg, HR 108 b.p.m., and lung crackles. The SaO2 was 98% with oxygen therapy (3 L/min). Biological results were NT-proBNP level, 21 627 pg/mL; creatininemia (122 µmol/L); and haemoglobin, 15.2 g/dL. Echocardiography revealed a dilated cardiomyopathy with global hypokinetic LV (LVEF at 10%), LA dilation (48 mL/m^2^), and increased LV filling pressure (restrictive mitral filling pattern with ratio E/lateral e′ at 22), systolic RV dysfunction (peak RV St velocity 5 cm/s and TAPSE 10 mm) with sPAP at 33 mmHg. The diaphragm ultrasound revealed a DE of 29 mm and a 2D diaphragm strain value of +18%. The patient received loop diuretics, ARA-II, and gliflozin. Six days later, a second echocardiography revealed an LVEF at 15% and a reduction of LV filling pressure (E/e′ 10) with RV systolic dysfunction (RV peak St velocity, 8 cm/s). The second diaphragm ultrasound revealed a DE of 8 mm (reduced) and a diaphragm strain value of −15%. The patient was discharged home at Day 11. After a 6-month follow-up, the patient remained stable without any re-hospitalization.

*Table 1* summarizes the case series of the study.

**Table 1:** Summary of data of the four cases

Patients	Parameters	Echo 1	Echo 2
Patient 1	Diaphragm excursion (mm)		
	Diaphragm 2D strain value (%)	−32	−3
	LVEF (%)	>50	>50
	E/lateral e′	7	
	sPAP (mmHg)	60	45
	NT-proBNP (ng/L)	15 436	10 721
Patient 2	Diaphragm excursion (mm)	51	7
	Diaphragm 2D strain value (%)	50	−1
	LVEF (%)	50	45
	E/lateral e′	13	
	sPAP (mmHg)	55	53
	NT-proBNP (ng/L)	3401	1711
Patient 3	Diaphragm excursion (mm)	6	5
	Diaphragm 2D strain value (%)		−2.4
	LVEF (%)	50	50
	E/lateral e′		
	sPAP (mmHg)	58	33
	NT-proBNP (ng/L)	2884	1660
Patient 4	Diaphragm excursion (mm)	29	8
	Diaphragm 2D strain value (%)	18	−15
	LVEF (%)	10	15
	E/lateral e′	22	10
	sPAP (mmHg)	33	
	NT-proBNP (ng/L)	21,627	3107

LVEF, left ventricular ejection fraction; sPAP, systolic pulmonary artery pressure; E/lateral e′, ratio between mitral E peak and lateral e’ peak via tissue Doppler imaging; NT-proBNP, N-terminal pro-brain natriuretic peptide.

## Discussion

The main findings are as follows:

1. The 2D diaphragmatic speckle imaging is feasible with high reproducibility.
2. It may be used to monitor diaphragm function in patients with AHF.
3. The diaphragm is frequently weak in patients with HF, even after decongestive therapy.

Respiratory muscle weakness is common in patents with CHF, reflecting the patient’s prognosis.^2,3^To our knowledge, this is the first case series to report the potential of ultrasound in assessing diaphragm function in patients in the CICU. The diaphragm strain value, as well as diaphragm motion, may be higher in AHF, and ultrasound can be used to monitor the respiratory status of patients under therapy. The persistently high diaphragm values may be related to the respiratory status of patients who had HF. In fact, for a given tidal volume achieved, patients with HF need larger swings in intrathoracic pressure than the healthy ones.^9^ Conversely, a reduced diaphragm motion may be related to diaphragm weakness that may affect HF prognosis.^2,3^ In this case series, Patient 3 who had diaphragm weakness experienced the onset of AHF 3 months later. As reported by Verissimo *et al.*,^10^ respiratory muscle weakness was highly prevalent among patients with AHF, with 76% of them (mean age, 75 years; mean LVEF, 33%) having a maximal inspiratory pressure of <70%. In addition, inspiratory muscle weakness is a prognostic biomarker in patients with HF.^2^ Thus, diaphragm ultrasound may be useful for stratifying patients with HF, in addition to the traditional prognostic biomarkers.

The DE measured with ultrasound strongly correlates with transdiaphragmatic pressure.^11^ In HF, diaphragm strength is reduced.^12^ In our study, after decongestive therapy, diaphragm weakness (diaphragm motion < 10 mm) occurred in patients with HF with preserved ejection fraction (Cases 2 and 3) and in one patient with HF with reduced ejection fraction (Case 4). Histologically, the proportion of slow fibres (fibre type I) of the diaphragm increases in HF.^13^ Diaphragm weakness in HF involves multiple pathophysiological factors. In patients with HF without pulmonary disease, the lung displays abnormalities with an obstructive or restrictive pattern.^14^ The increase of the LA pressure induces (*via* a backward transmission) hydrostatic pressure with pulmonary system congestion, which causes lung compliance reduction and ultimately lung stiffness. This feature provokes reduction of the DLCO (diffusing capacity of the lungs for carbon monoxide) in relation to alveolocapillary membrane derangement and reduction in lung volume measured using spirometry.^12^ Bronchial oedema may also occur in patients with HF, providing an obstructive respiratory pattern. Finally, a lower systolic BP and a reduced LV function, as well as cytokine derangement, may be involved in respiratory muscle weakness.^15^

During the act of breathing, the respiratory muscles are solicited to provide negative pleural pressure, owing to the entry of air in the lungs. Normally, during inspiration, the muscle fibres of the diaphragm shorten as the diaphragm contracts, proving caudally displacement of the diaphragm.

In AHF, the respiratory system’s elastic properties change and/or its resistive properties increase. Hence, inspiratory muscles including the diaphragm have to overcome the load, thereby increasing the muscular strain. Our case series illustrate this phenomenon with a higher strain diaphragm value in patients with AHF. A diaphragm-positive strain may be related to passive stretching resulting from higher thoracic loading.

A higher resistive load may induce diaphragm injury, as reported in animal models with high resistive load.^16^ In patients with acute dyspnoea, high inspiratory efforts can induce higher intrathoracic pressure swings, which are potentially deleterious for the lung (patients’ self-inflicted lung injury).^17^ Thus, monitoring diaphragm function may help screen patients who may need NIV to reduce to risk of myotrauma.^16^

Respiratory effort may be assessed using the 2D diaphragmatic strain imaging because diaphragm strain is strongly associated with transdiaphragmatic pressure.^5^ This imaging tool is angle independent. The 2D diaphragmatic speckle tracking echo has been validated by Oppersma *et al.*^5^ who showed a strong association between diaphragm effort and the diaphragm strain value.

This study is limited by its type, that is, case series. Two-dimensional strain imaging software has been validated to assess myocardial function. Specific software’s focus on respiratory cycle needs to be elaborated for research.

## Conclusion

Monitoring diaphragm muscle effort using ultrasound may be clinically relevant, considering that high respiratory efforts can induce diaphragm injury. Ultrasound can be used to search for diaphragm weakness in patients with HF and those with HF concurrent with respiratory diseases.

## Supplementary Material

ytae632_Supplementary_Data

## Data Availability

Data are available on a reasonable delay from the author.

## Lead author biography

Dr Abdallah Fayssoil is an associate professor in cardiology in the University of Versailles Saint Quentin en Yvelines, in France.

## Notes

**Consent:** The authors confirm that consent for submission and publication of this case report including image(s) and the associated text has been obtained from patients in line with the COPE guidelines.

**Funding:** None declared.
